# Implications of lifestyle factors on male reproductive health

**DOI:** 10.5935/1518-0557.20240007

**Published:** 2024

**Authors:** Damilare E. Rotimi, Shio Kumar Singh

**Affiliations:** 1 SDG 03 Group - Good Health & Well-being, Landmark University, Omu-Aran 251101, Kwara State, Nigeria; 2 Department of Biochemistry, Medicinal Biochemistry, Nanomedicine & Toxicology Laboratory, Landmark University, PMB 1001, Omu-Aran-251101, Nigeria; 3 Department of Zoology, Institute of Science, Banaras Hindu University, Varanasi 221005, India

**Keywords:** infertility, lifestyle factors, sperm, testis

## Abstract

In recent decades, there has been a substantial decline in sperm quality in humans, with lifestyle factors playing a major role in this trend. There are several lifestyle factors which are contributing to male infertility. This review, however, discusses factors such as obesity, diet/nutrition, psychological stress, radiation exposure, cigarette smoking, and alcohol use with reference to male infertility. Sperm count, motility, morphology and sperm DNA may be adversely affected by lifestyle factors, which may also affect the endocrine regulation of reproductive function. The decline in male fertility has a significant impact on fertility rates, and the resulting implications for the human population make this a serious public health concern in the twenty-first century. Thus, lifestyle interventions through a specific framework of educational, environmental, nutritional/physical exercise, and psychological support coupled with the use of nutritional antioxidants supplements can help couples achieve better health and well-being and improve their fertility prospects or increase their chances of conception.

## INTRODUCTION

Infertility is the inability of couples to conceive after at least 12 months of unprotected sexual intercourse. It affects around 10 - 15% of couples, with an estimated 186 million people worldwide. The male factor has been identified as the primary cause of infertility in 20 to 30% of cases, and as a contributory cause in 50 to 60% of couples (Rahban & Nef, 2020). Male infertility disorders is commonly associated with oligospermia (low sperm volume), teratozoospermia (abnormal sperm morphology) or azoospermia (no sperm production at all) (Abarikwu *et al.*, 2020; Kumar & Singh, 2015; Rahban & Nef, 2020). It is reported that semen quality has been steadily declining for several decades. The concentration of sperm in fertile men and in men of unknown fertility was examined in a systematic review that spanned nearly 40 years (Carlsen *et al*., 1992). Sperm concentration dropped from 113 to 66 million/ml, and semen volume decreased from 3.40 to 2.75 ml during the period of study (Carlsen *et al*., 1992). A study conducted by Splingart *et al*. (2012) between 1976 and 2009 examined changes in the quality of sperm over time and found a significant reduction in the total sperm count (from 443 to 300 million), motility (from 64 to 49%), and vitality (from 99 to 80%). Furthermore, sperm with normal morphology also decreased from 67 to 26%.

Causes of male infertility include advanced paternal age, exposure to chemicals, medical conditions such as sarcoidosis, anatomical anomalies, physical obstruction following a vasectomy, and genetic anomalies. Male fertility is now negatively impacted by environmental, occupational, and lifestyle factors (Mishra *et al*., 2012). Among the many lifestyle factors, unhealthy habits that lead to obesity (sedentary lifestyle & poor nutrition), using electronic devices that emit electromagnetic radiation (cell phones, laptop computers), stress, alcohol consumption,and smoking are some of the common factors that cause male infertility. Obesity in males has been associated with lower ejaculate volume, a higher risk of sperm DNA damage, and a higher incidence of azoospermia or oligospermia.

Similar effects have also been seen in males following excessive drinking and smoking. The use of alcohol causes testicular shrinkage, a decline in libido, and changes in semen parameters in men, while smoking is linked to reduced semen quality. The aforementioned information, thus, implies that infertile persons might adopt nonmedical measures to increase their chances of conceiving naturally or via assisted reproductive technology (ART).

However, studies have shown that a large number of men taking fertility treatments frequently have unhealthy lifestyles, which may reduce their likelihood of conceiving (Qiao & Feng, 2014; Zeinab *et al*., 2015). Therefore, this review focuses on impact of above-mentioned lifestyle factors on male fertility along with the mechanisms of their actions and the treatment options available. For this purpose, a literature search was performed with the use of bibliographic databases of peer-reviewed journals (Scopus, Google Scholar, Science Direct, and Web of Science). Relevant articles were identified by keywords and medical topic search terms: “male infertility”, “obesity”, “alcohol consumption”, “diet”, “stress”, “drug abuse”, “cigarette smoking”, and “mobile phone”. Pertinent studies that were published from the January 2000 until December 2022 were included (over 217 articles), and the relevant literature from the articles’ references was also examined. Only English language studies reporting on the lifestyle factors associated with male infertility were considered, while non-English language studies and those without a published abstract were not considered.

## LIFESTYLE FACTORS AND MALE INFERTILITY

**Figure 1:**
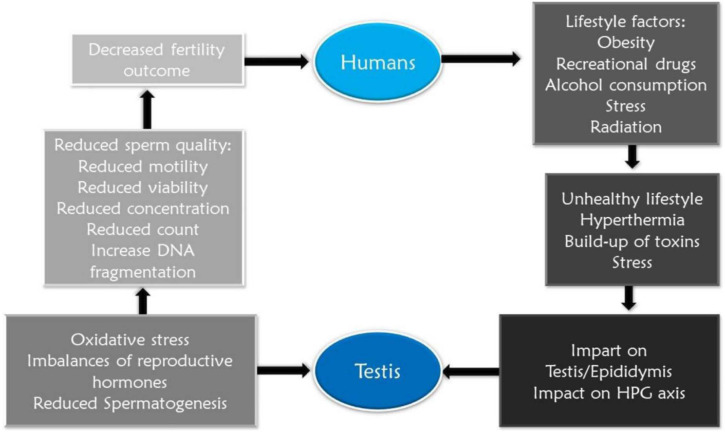
Summary of the effect of lifestyle factors on male reproductive health

Lifestyle factors are modifiable behaviors and practices that can affect an individual’s overall health and well-being. Researchers are becoming increasingly interested in exploring the significance of lifestyle factors in the etiology of infertility. There is a strong correlation between infertility and a wide range of lifestyle behaviors including drinking alcohol, obesity, eating fat-rich diets, smoking, stress, as well as drug misuse (Figure 1). It is, however, possible to restore normal oocyte maturation in women and to increase semen quality in men by adjusting specific lifestyle factors. Due to advances in assisted reproductive technology, most infertility issues may be treated with major procedures (Emokpae & Brown, 2021). Understanding the many processes through which modifiable lifestyle choices impact male infertility would be very effective in managing the affected individuals.

### Obesity

The state of being overweight is known as obesity. To be considered obese, a person’s body mass index (BMI) must be at least 25 kg/m^2^. There has been a dramatic rise in obesity rates in recent times. For instance, the rate of obesity in the United States is above one-third of the population (Ogden *et al*., 2010). For young adults who are of reproductive age, the obesity rate has quadrupled during the 1970s to approximately 2.1 billion persons worldwide (Rippe, 2021). Men who are overweight or obese have an estimated 1.1- to 1.4- fold greater risk of sub/in-fertility. An epigenetic modification of sperm DNA has been linked to obesity-related reproductive problems, as have other possible causes, such as a testosterone (T) deficiency-related influence on sperm or other hormonal changes (Liu & Ding, 2017).

Overweight and obese males had reduced sperm count rates, sperm concentration, and low ejaculate volume as measured by a population-based study. According to a meta-analysis of 21 studies (n=13,077), it was concluded that obesity was related to an increased risk of oligospermia and azoospermia, respectively (Kahn & Brannigan, 2017). Another study revealed that increased BMI in males reduces sperm concentration and motility and impairs the integrity of the sperm DNA (Chavarro *et al*., 2010).

Men who are overweight are three times more likely to have a sperm count of less than 20 million/ml than men with a healthy weight, and this indicates oligozoospermia (McCray *et al*., 2020). Men with a greater BMI (>25 kg/m^2^) had a lower total sperm count compared to men with normal BMI weight (Chavarro *et al*., 2010). Total sperm count and concentration were negatively correlated with body mass index in a large-scale investigation of 1558 males in the Danish military (Jensen *et al*., 2004). Although the mechanism by which obesity reduces sperm motility and morphology remains unknown, it is known that obesity negatively affects overall fertility.

Several reports have suggested that the hypothalamic-pituitary-gonadal (HPG) axis is affected by obesity (Kahn & Brannigan, 2017; Shpakov *et al*., 2018). Obesity leads to hormonal imbalance directly or indirectly as it affectssex hormone-binding globulin (SHBG)and decreases the levels of free testosterone as well as follicle-stimulating hormone (FSH) and luteinizing hormone (LH) which ultimately impact spermatogenesis (Shpakov *et al*., 2018). Hypogonadotropic-hypoestrogenic-hypogonadism is caused by the aromatization of steroids to estrogens in peripheral tissues, which results in a significant drop in testosterone levels and a rise in estradiol (Corradi *et al*., 2016). To emphasize the negative feedback impact of higher total estradiol, Stellato *et al*. (2000) found that reduced sex hormone-binding globulin was observed in obese males as a result of hyperinsulinemia.

Several studies have revealed that obesity has an impact on spermatogenesis and Sertoli cell activity, as evidenced by the considerable decrease in inhibin B concentration compared with the decline in FSH levels (Leisegang *et al*., 2021; Saikia *et al*., 2019). Reports show that overweight males had lower levels of inhibin B and higher amounts of estradiol. Moreover, increased testosterone-to-estrogen conversion occurs in obese persons because of the existence of white adipose tissue, which also impacts the HPG axis, thus reducing gonadotrophin secretion. In the long run, this causes secondary hypogonadism and reduces sperm production. The white adipose tissue causes a decrease in testosterone synthesis due to an increase in leptin production (Leisegang *et al*., 2021).

Furthermore, scrotal adiposity induces testicular heat stress that results eventually in oxidative stress as adipokine enhances reactive oxygen species (ROS) production, resulting in the inhibition of spermatogenesis. Obese males have lower sperm counts along with an unbalanced ratio of oxidants to antioxidants. Apoptosis, sperm motility, DNA integrity, and sperm-oocyte interaction are all affected by increased oxidative stress (Durairajanayagam *et al*., 2015).

The number of live births per ART cycle among obese men who attempt ART is lower. Weight gain during ART treatment may harm the reproductive outcomes of obese men, including reduced blastocyst development, pregnancy, and live birth rates, and the increased need for intracytoplasmic sperm injection (ICSI) use has been linked to increased paternal BMI (Bakos *et al*., 2011). Although male partner BMI was linked with decreased sperm concentration, motility, and morphology, it was not associated with later changes in fertilization, implantation, or pregnancy rates in a group of 250 men who had ICSI (Bakos *et al*., 2011).

### Diet

Diet and nutrition can be classified generically as general dietary patterns or more precisely as vitamin/mineral supplements. Reports show that the diet affects semen quality and male reproductive capacity independently (Gaskins *et al*., 2012; Nilsson *et al*., 2023). The so-called western diet has become the primary nutritional model for both developing and developed countries in recent decades. Animal proteins, saturated and trans fatty acids, and simple carbohydrates make up a large portion of the typical Western diet, while dietary fiber and essential unsaturated fatty acids are severely lacking. It is a high-calorie, pro-inflammatory diet which is also low in nutrients (Nilsson *et al*., 2023). As the western diet model has become more popular, it is obvious that the parameters used to judge the quality of semen have declined (Ferramosca & Zara, 2022). Poorer semen parameters and lower fertility have been linked to a diet high in processed foods (Ferramosca & Zara, 2022; Nilsson *et al.*, 2023). However, an increased risk of asthenozoospermia, oligospermia, and teratozoospermia may occur as a result of this diet, with reduced sperm quality (Oostingh *et al*., 2017). The structure of spermatozoa, as well as the health of offspring and future generations is negatively impacted by a high-fat diet and obesity. Sugar, soy products, processed meat, red meat, saturated and transfatty acids, potatoes, dairy, and increased caffeine and alcohol use are all highly linked to male infertility in men. Asthenozoospermia may be linked to increased insulin resistance and oxidative stress as a result of sweet consumption. Reduced sperm concentration and poor motility have been linked to high carbohydrate diets and refined sugars (Attaman *et al*., 2012). Red meat consumption, which is high in saturated fatty acids, has been linked with decreased sperm count and motility in a dose-dependent manner. An increase in dairy consumption has been suggested to have an increased risk of oligoor asthenozoospermia (Oostingh *et al*., 2017).

Semen quality can be affected by a reduced diet of fruits and vegetables, fiber, and polyunsaturated fatty acids (especially omega-3 fatty acids), and foods rich in micronutrients, antioxidants, and phytochemicals (especially vitamins C and E, selenium, β-carotene, L-carnitine, zinc, and lycopene) (Giahi *et al*., 2016).

Increased testicular and seminal oxidative stress as well as sperm DNA fragmentation and decreased chromatin condensation are all linked to poor dietary intake. For example, an experimental study showed a decrease in the height and diameter of the seminiferous epithelium and seminiferous tubules in mice fed a high-fat diet leading to long-term reproductive and metabolic changes (Frihauf *et al*., 2016; Giahi *et al*., 2016). Furthermore, sperm viability, concentration, and DNA integrity were also reduced.

Male-factor infertility can be diagnosed and treated using diet, however, the mechanisms of action is unknown. It is known that increasing the consumption of fruits, vegetables, legumes, and fish may improve reproductive health. It was shown that males who ate a diet rich in fruits, vegetables, and legumes had superior sperm quality and less fragmentation in the sperm cell than those who did not eat these items regularly (Kayode *et al*., 2020a).

A cross-sectional study from the University of Rochester on 188 young males aged 18-22 revealed similar findings (Giahi *et al*., 2016). The research indicated that men who ate a “prudent” diet (high intake of whole grains, legumes, vegetables, fruit, chicken, and fish) had higher progressively motile sperm counts than those who ate a “Western” diet (high intake of red and processed meat, high-energy pizza, snacks, drinks, refined grains, and sweets). There were no variations in sperm concentration or morphology between the diets. Semen parameters may be linked to the type and volume of meat consumed (Hartvigsen *et al*., 2007).

The consumption of processed meat was negatively connected with sperm morphology in a prospective analysis of 155 infertile couples by Afeiche *et al*. (2014), who observed a 1.7% drop in sperm morphology.

Poor diet are linked to abnormal semen quality and results in higher risk of infertility, and oxidative stress is the primary mechanism by which these relationships are formed (Ferramosca & Zara, 2022). Alpha-lipoic acid, selenium, cobalamin, and beta-carotene have essentially been used in sustaining male factor fertility in several studies. Many have proposed that multivitamin supplementation may play a role in enhancing male fertility. There were 48 randomized controlled trials (RCTs) in which 4179 men with infertility were assessed for improvements in live birth rate, pregnancy, and semen parameters (Zhao *et al.*, 2013). Vitamins E and C, zinc, polyunsaturated fatty acids, folate, carotenoids, ubiquinol, and pentoxifylline were among the antioxidants included in the study. Antioxidant supplementation has been shown to boost live birth rates and pregnancy rates, although its effect on miscarriage rates remains uncertain (Beigi Harchegani *et al*., 2020; Ferramosca & Zara, 2022; Kayode *et al*., 2020a).

### Stress

Stress could be psychological, social, physical, and biological. A wide range of stressors may be found in everyday activities like job and family commitments as well as life events and unexpected unpleasant lifestyle shifts. The prevalence of stress in today’s society may be assumed to be high, with many people experiencing a differentform of stress. This is common in couples who are trying to conceive naturally but are unable to. Reduced paternity and aberrant semen characteristics are linked to psychological stress, suggesting that stress contributes to male infertility (Ghezzi *et al*., 2018; Zorn *et al*., 2008).

The male reproductive potential may be negatively affected by stress in a variety of ways. Scrotal hyperthermia-induced genital heat stress is a substantial contributor to male infertility. Testicular heat stress can be caused by cryptorchidism, varicocele, prolonged sitting, and exposure to radiant heat. Spermatogenic arrest, sperm DNA damage, oxidative stress, and germ cell death are all consequences of elevated scrotal temperature (Durairajanayagam *et al*., 2015). Increased heat production in the testis is linked to cycling as a sport. Following 16 weeks of intense cycling training in young, healthy male road riders, ROS and malondialdehyde levels were found to increase, while total antioxidant capacity and enzymatic antioxidants were decreased. Even after four weeks of recuperation, these alterations persisted. Despite the 4-week recuperation period, seminal interleukin levels were elevated and sperm parameters decreased in these nonprofessional cyclists (Maleki *et al*., 2014).

The sympathetic nervous system is activated and this affects the hypothalamus-pituitary-adrenal (HPA) axis during the classical stress response. Inhibition of the HPG axis and testicular Leydig cells is mediated via the HPA axis and gonadotrophin inhibitory hormone (GnIH). As a result, the HPG axis is inhibited which affects testosterone level and the spermatogenic process. Psychological stress affects spermatogenesis which could be as a result of decrease in testosterone production. Increased levels of corticosterone have been shown to inhibit testosterone and inhibin in stressed rats (McGrady, 1984). The impact of psychological stress on sperm quality and reproductive hormones of infertile men has been investigated. The Hospital Anxiety and Depression score (HADS) questionnaires were used to determine the amount of psychological stress, and 27% of the population experienced psychological stress. Higher levels of FSH and LH were found in males with a high degree of stress than in those with moderate level of stress. Despite this, GnRH levels remained stable. Concerning sperm count and motility, men with HADS 8 had significantly lower sperm counts compared to those with normal HADS. Sperm motility and count were favorably connected with serum testosterone levels, although LH and FSH were negatively correlated (Bhongade *et al*., 2015). Acute restraint stress was found to inhibit sperm motility in animal model research after 30 minutes of restraint. Adrenocorticotrophic hormone (ACTH) and corticosterone levels were raised, but FSH, LH, and testosterone levels were reduced. According to the results, the enhanced HPA axis activity slowed down the HPG system.

Gollenberg *et al*. (2010) examined the effects of stress on total sperm counts, sperm density, morphology, and forward motility in 950 males. Stress was found to have a substantial impact on sperm quality. Gonadal activity is disrupted and testosterone and LH pulses are reduced, which in turn affect spermatogenesis and sperm characteristics. After a diagnosis of infertility, follow-up consultations and failed *in vitro* fertilization (IVF) procedures are reported to elevate the stress level.

Psychological stress in terms of work, personal, and family stress and the semen quality were examined. Workplace stress had a negative impact on sperm quality, but with a positive association between stress and sperm DNA damage. Satisfaction with family life is inversely related to the percentage of motile sperm cells. Another study looked at the effects of stress on sperm quality in their jobs and stressful life events. Semen quality, particularly sperm motility and the proportion of normal morphology, was negatively correlated with perceived stress and stressful life events (Jurewicz *et al*., 2014).

Several studies have shown that increased temperature can affect fertility by altering sperm parameters (number, motility, and morphology) as well as by damaging sperm membrane integrity (Jung *et al*., 2008; Sabés-Alsina *et al*., 2016). The thermoregulation of the scrotal sac is an essential defense mechanism because it reflects the temperature of the testicles. Damage to the sperm DNA breakage and plasma membrane of both mitochondrial and nuclear genomes is caused by increased ROS production at higher temperatures. This results in apoptosis and cellular damage. Toxic effects of testicular heat stress have been reported; heat compromise sperm DNA and triggers apoptosis in both the intrinsic and extrinsic apoptotic pathways in mice (Wechalekar *et al*., 2010).

### Radiation

There has been an exponential rise in the number of people using cell phones in the last decade, raising questions about the potential dangers of the high-frequency electromagnetic field (EMF) radiation emitted by these devices and its potential implications for human health. The phone emits EMF, that is low-level Radio Frequency (RF-EMF) at 850 MHz - 2.4 GHz and can be absorbed by humans (Agarwal *et al*., 2008). Depending on the amount of exposure time, most radiation have detrimental effects on the sperm. With more than 6.8 billion people worldwide with mobile phone subscriptions, it is said that an average person uses a phone for 90 minutes a day, equivalent to nearly four years of their life spent staring at a phone screen. Due to the high prevalence of mobile phone usage, it is crucial to understandthe correlations between RF-EMR and semen quality (Levis *et al*., 2015).

A specific absorption rate (SAR) of 2.0 W/kg is the legal limit for mobile phones; nevertheless, most phones have an average SAR of just 1.4 W/kg. Because every phone is unique and has a unique SAR, it is impossible to compare the radiation emitted by them all, although studies have revealed certain associations (Adams *et al.*, 2014). The closer the mobile phone is to testis, the more electromagnetic radiation exposure (Adams *et al*., 2014). For instance, some studies suggest that keeping mobile phones in the shirt pocket causes more harm, placing the phone in a jeans pocket closer to the testis can result in infertility, and placing it closer to the brain might harm the brain tissue (Takebayashi *et al*., 2008). According to a study, phone calls for more than an hour a day, or while it is charging, has been linked to lower sperm concentrations (Zalata *et al*., 2015). Another research revealed that males exposed to mobile phone radiation showed an 8% reduction in sperm motility compared to those not exposed to radiation (Rehman *et al*., 2018). Males who carry a mobile phone in their pants pocket have a statistically significant increase in DNA fragmentation compared to those who keep their phone in their shirt pocket (Rehman *et al*., 2018).

Portable computers are also a source of low-level radio-frequency electromagnetic fields (RF-EMF) (laptops, connected to local area networks wirelessly, also known as Wi-Fi). People in their 20s and 30s are increasingly using laptop computers. It is often the case that laptops are linked to the internet through Wi-Fi and that they are placed on the lap near the testicles (the emission of electromagnetic waves is 7-15 times higher beneath the laptop than under the basal situation without a portable computer). Moreover, the high temperatures generated by computers can alter spermatogenesis by raising the scrotal temperature (Durairajanayagam *et al*., 2015). Experiments on rats have confirmed that exposure to mobile phone RF-EMF induces histological alterations in the testis. According to a study by Oh *et al*. (2018) prolonged exposure to 4G-LTE-based electromagnetic fields (EMFs) affects rat spermatogenesis. Mobile phone usage for lengthy periods, such as 18h per day, caused lower sperm and Leydig cell counts in males, particularly those who are in their reproductive years (Oh *et al*., 2018).

The male reproductive system is a complex, multi-part structure that relies on the coordination of both external and internal factors. Cell phone EMF-induced damage to spermatozoa has been linked to ROS. The sperm plasma membrane is rich in polyunsaturated fatty acids (PUFA), making it extremely vulnerable to lipid peroxidation caused by ROS. Spermatozoa membrane integrity and motility are compromised by lipid peroxidation. The RF-EMR also caused DNA damage, which was believed to accelerate sperm cell death and promote testicular carcinogenesis. About 124 semen samples were exposed to 1h of mobile phone radiation in another *in vitro* investigation, with the results recorded before and after the exposure (Zalata *et al*., 2015). An increase in DNA fragmentation and an overall reduction in sperm motility, sperm linear velocity, and sperm acrosome reaction were found. Another research on 32 healthy men who had their sperm samples exposed *in vitro* for 5h validated these findings (Gorpinchenko *et al*., 2014). The number of sperm with progressive motility decreased and the proportion of sperm with DNA fragmentation increased dramatically in the exposed samples. Data from ten research papers involving 1492 samples were reported (Adams *et al*., 2014). The impacts on sperm motility and viability were largely linked to cell phone use, although the effects on concentration were more equivocal. An increase in DNA breakage and a reduction in sperm motility were linked to the usage of wireless network-enabled laptop computers (Adams *et al*., 2014).

It has been claimed that RF-EMF can alter sperm motility by increasing superoxide anion concentrations due to an elevated ROS level. The free radicals produced by sperm mitochondria reduce vitality and mobility as they oxidize membrane phospholipids. Antioxidant enzymes like glutathione peroxidase (GPx) and superoxide dismutase (SOD) have their activity reduced by the ROS produced by electromagnetic fields. It triggers apoptosis, DNA damage, and changes in the expression of cytochrome c, Bax, and other genes, and it has far-reaching effects on the integrity of both mitochondrial and nuclear genomes. A substantial increase in DNA fragmentation and reduction in sperm motility were seen in experiments in which human spermatozoa were exposed to a wireless internet laptop (Agarwal *et al*., 2009).

### Use of recreational drugs

The use of drugs for recreational purposes may have a number of unfavorable health effects. Certain molecules that interact with the cannabinoid receptor, such as phytocannabinoids, and especially the high-potency synthetic cannabimimetics, have been linked to cardiotoxic (e.g., dysrhythmias, cardiac arrest, chest pain, and myocardial infarction) effects (Nwonuma *et al*., 2021).

Tobacco, cannabis, and alcohol have all been linked to infertility (Fronczak *et al*., 2012). Direct cytotoxic effects, altered cortisol levels, altered GnRH secretion, testicular atrophy, hyperprolactinemia, increased oxidative stress, increased testosterone aromatization, and sperm hyperactivation are a few of the possible mechanisms for changes in semen parameters caused by substance abuse (Emanuele & Emanuele, 1998; Fronczak *et al*., 2012). Despite various research findings, there is a paucity of information on abstinence from drugs or alcohol usage on the restoration or improvement of fertility.

Smoking has a deleterious influence on spermatogenesis as well as on semen quality (Rehman *et al*., 2018). The deleterious effects of smoking on semen parameters were confirmed in a meta-analysis study (Li *et al*., 2011). It was found that smoking was associated with decreased semen volume (-0.25 mL), total sperm count (-32.2 million/mL), concentration (-7.1 million/mL), normal morphology and motility (1.9%) in all 57 studies (29,914 participants) that examined the impact of lifestyle factors on male infertility. Smoking was found to be an independent risk factor (-4.9%) (Li *et al*., 2011). An ultrastructural examination of sperm from a small group of otherwise healthy smokers (n=62) was undertaken by Joo *et al*. (2012). Although electron microscopy revealed no abnormalities, individuals who smoked more than 20 cigarettes a day had lower sperm counts than the rest of the group (Joo *et al*., 2012).

Acute effects on LH and FSH as well as on T production and spermatogenesis have been found to occur when cannabis tetrahydrocannabinol, the primary psychoactive component of marijuana, is consumed (Daniell, 2002). Additionally, cannabis appears to directly impair sperm function, motility, and viability. The cannabinoid receptors (CB1 and CB2) have been identified as significant regulators of sperm activity, even though the underlying mechanism has not been thoroughly deciphered.

In all forms of alcohol abuse, there is an increase in estrogen and a reduction in testosterone (Bansal, 2013). Another lifestyle risk linked to low semen quality is excessive alcohol usage. In the Western world, particularly in Europe, it is a regular occurrence. In reality, the UK is the world’s top consumer of alcoholic beverages. Alcohol dependency affects around 17.6 million individuals, and binge drinking is practiced by millions. As a result, it has been established that there is a dose-dependent link between alcohol intake and semen quality (Jensen *et al*., 2014a; 2014b). Young guys of reproductive age are also reported to consume the most alcohol in the shortest time.

It has been shown in a study of 1221 Danish males that regular alcohol drinking has negative effects on semen (Jensen *et al*., 2014a). The patients in this study were followed for a longer length of time, which might indicate more accurate results. Sperm quality was found to be negatively impacted by even a moderate amount of weekly alcohol use of 5 units. Semen, on the other hand, was significantly affected by the ingestion of 25 units per week. This sort of drinking has been linked to decreased testosterone and SHBG levels (Jensen *et al*., 2014b). There is evidence that drinking damages Leydig cells and impairs the HPG axis, which can contribute to decreased testosterone levels in males. As sperm production and quality depend on the proper balance of hormones, each of these events might have an unfavorable effect on sperm quality. For the first time in over 30 years, studies on the quality of sperm and the hormonal issues that go along with it in alcoholics were published. Over half of the men whose bodies were examined for alcohol-related death exhibited spermatogenic arrest, according to the findings of the autopsy. Alcohol consumption and its link to semen quantity, sperm morphology, and motility were reported to be linked in a meta-analysis (including 29,914 individuals) in 2011 (Li *et al*., 2011).

## MECHANISM INVOLVED IN LIFESTYLE FACTORS AND MALE INFERTILITY

In the testis, a multiphase cellular process known as spermatogenesis continuously produces spermatozoa. In the seminiferous tubules, spermatogonia develop into primary and secondary spermatocytes, spermatids, and spermatozoa. The development, regulation, and physiologic functioning of sperm requireseveral hormones essential for spermatogenesis (Kayode *et al*., 2020a; 2020b). Gonadotropin-releasing hormone (GnRH) from the hypothalamus stimulates the anterior pituitary to release FSH and LH; FSH acts on the seminiferous epithelium, while LH exerts its effects on spermatogenesis via testosterone, which is secreted by Leydig cells under the influence of LH. Thus, LH, testosterone and FSH are the key players that regulate spermatogenesis. Growth hormone and insulin-like growth factor 1 may also play a role in the regulation of spermatogenesis by acting in a paracrine fashion. Spermatogenesis depends on high amounts of testosterone and dihydrotestosterone (DHT) in the testicles as they both play a major role in developing male sexual characteristics during the embryonic and pubertal phases (Olaolu *et al*., 2018).

The spermatogenic process has been negatively impacted by the different lifestyle factors either directly or indirectly (Table 1). Mitotically active spermatogonia may be inhibited or damaged by lifestyle-related factors via multiple mechanisms. Examples of mechanisms involved in lifestyle factors-linked to fertility include ROS generation, inflammation, testicular apoptosis, impaired gonadal secretion and impaired spermatogenesis. For instance, during processes involved in sperm production (acrosomal response, capacitation, and fertilization), ROS are generated and could be implicated when its level is high. Thus, ROS promotes oxidative stress, which negatively impacts spermatogenesis and sperm function when present in excess (Singh & Singh, 2019). Oxidative stress could induce cellular apoptosis and reduce acrosomal activity of cells. ROS damages lipid membranes in the seminiferous tubules via peroxidation, an action magnified by the low cytoplasmic concentration of spermatozoa (Rotimi *et al*., 2022). As a result of the decreased adenosine triphosphate availability caused by increased ROS levels and DNA damage, sperm cells are unable to reach the oocytes to achieve fertilization. Thus, natural fertility as well as ART could be directly influenced by ROS.

**Table 1:** Summary of lifestyle factors and mechanisms involved in male infertility.

Lifestyle factors	Proposed mechanisms	References
Obesity	Epigenetic modification of sperm DNA Reduced spermatogenic function Impaired sperm DNA integrity Suppressed HPG axis Increased oxidative stress	Khodamoradi *et al*. (2020)
Mobile phone use/radiation	Impaired sperm function, motility, and viability Increased sperm DNA fragmentation Reduced sperm anti-oxidative activities	Durairajanayagam (2018)
Diet/Nutrition	Poor semen quality Compromise sperm DNA integrity Increased oxidative stress	Nilsson *et al*. (2023)
Stress	Impaired sperm function, motility, and viability Suppressed HPG axis Increased oxidative stress	Bhongade *et al*. (2015)
Recreational Drugs	Proposed mechanisms	References
Tobacco	Impaired spermatogenesis	Nwonuma *et al*. (2021)
Marijuana	Impaired spermatogenesis and steroidogenesis	Gundersen *et al*. (2015)
Cannabis	Reduced gonadotropic hormones (LH and FSH) Impaired sperm function, motility, and viability	Nwonuma *et al*. (2021)
Opioids	Increased sperm DNA fragmentation Reduced spermatogenic function Suppressed HPG axis Impaired acrosomal reaction	Gundersen *et al*. (2015)
Anabolic steroids	Impaired spermatogenesis and steroidogenesis	McBride & Coward (2016)
Alcohol	Reduced T, estradiol, and SHBG levels Increased oxidative stress Poor semen quality and spermatogenic arrest	Nwonuma *et al*. (2021)

## PROMOTION OF HEALTHY LIFESTYLE BEHAVIORSAS A MANAGEMENT/TREATMENT OPTION

The beneficial modifications to habits, behaviors, and conditions that have in the past adversely influenced fertility constitute the healthy lifestyle practices promoting fertility. Since maintaining and promoting fertility mostly depend on one’s lifestyle, people must examine their unhealthy behaviors and control them by deciding on more appropriate conduct (Faure *et al.*, 2014). Most infertile people want to succeed in their treatments and adjust their behavior in ways that could increase their fertility. Healthy living practices include stress management, interpersonal interactions, mental growth, physical exercise, and diet (Leisegang *et al.*, 2021).

Selective lifestyle choices are affordable and helpful as tools in the treatment and prevention of male infertility. Male infertility can be affected by a healthy diet and regular exercise, thus excellent nutrition and a well-balanced diet should be promoted (Leisegang *et al*., 2021; Mir *et al*., 2018). Diet and exercise have been shown in clinical studies to enhance sperm characteristics such as concentration, motility, morphology, and DNA fragmentation in men with decreased adiposity, even if BMI remains the same (Faure *et al*., 2014; Mir *et al*., 2018). Alternatively, bariatric surgery is a successful weight-loss therapy. Despite correcting the reproductive hormone profile, it may not have an impact on sperm function within two years following the procedure. Embryo growth and metabolic function are all improved in the children of obese parents who lost weight (McPherson & Lane, 2015). Semen quality improves as a result of adherence to the Mediterranean diet or comparable dietary trends. Diets high in fruits, vegetables, fiber, seafood, nuts, seeds, and vegetable oils, as well as foods high in antioxidants, fall under this category. Ascorbic acid, tocopherols, selenium, zinc, L-arginine, and coenzyme Q10 are micronutrients that are particularly favorable to male fertility, as are carotenes (Giahi *et al*., 2016).

Antioxidants are essential in managing and treating male infertility. Other examples include compounds like ferritin and albumin, or enzymes like SOD and catalase. Excess ROS in the seminal ejaculate can be reduced and converted to molecules that are less harmful to cells by these antioxidant enzymes. A high concentration of ROS increases oxidative stress, which damages sperm proteins, lipids, and DNA leading to sperm malfunctioning. For instance, ascorbic acid is a well-known antioxidant that protects the testicles from oxidative damage. As a result of its ability to retain this antioxidant in an active state, it contributes to the maintenance of spermatogenesis (Nayanatara *et al*., 2008). Dehydroascorbate reductases, which are many in the testis, are responsible for maintaining vitamin C levels in a reduced condition (Nayanatara *et al*., 2008). After being transformed into cysteine (a precursor to glutathione), N-Acetyl cysteine (NAC) has antioxidant effects. NAC improved sperm motility *in vitro* by reducing ROS levels and thereby enhancing germ cell survival (Oeda *et al*., 1997). Clinical research on antioxidants has often yielded mixed outcomes. A randomized study on 468 infertile males with idiopathic oligo-asthenoteratospermia found that N-acetyl-cysteine and selenium treatment had a positive impact (Safarinejad & Safarinejad, 2009). Men who take oral antioxidants appear to have a slightly higher live birth rate than those who do not, according to a Cochrane meta-analysis of 33 studies (Safarinejad & Safarinejad, 2009).

In general, lifestyle factors may be improved by exercising, which lowers oxidative stress and DNA fragmentation. Men’s fertility has been demonstrated to increase with resistance training (Maleki *et al*., 2014). Conscious living and enough sleep are essential for overall health, which also impact reproductive health. However, the interaction between sleeping habits and reproductive hormones affects fertility in both directions, making it more complicated. Finally, avoiding harmful lifestyle choices like smoking and alcohol intake might improve the results of male infertility. Due to the disruption of the reproductive processes, most illicit and prescribed drugs have a significant influence on fertility. Regarding improving altered sperm parameters, complete quitting of tobacco is necessary, whereas smoking cessation has been linked to better sperm parameters. There is some evidence that moderate and excessive alcohol intake (>25 units per week) should be avoided as a safe lifestyle practice (Gaur *et al*., 2010; Sansone *et al*., 2018). Consuming more than three cups of caffeine per day might lead to health complications. The use of cannabis should also be avoided, especially regarding infertility. Off-label usage of anabolic steroids can negatively affect male hypothalamus-pituitary-thyroid (HPT) axis modulation, including aromatase inhibitors, gonadotropins, and estrogen receptor modulators.

Infertility can be prevented by limiting exposure to electromagnetic waves emitted by technological devices like cell phones. Although it is impossible to completely remove all environmental risks, steps may be taken to minimize their impact. The fecundity of males has been shown to improve with the use of mind-body stress reduction techniques like meditation and yoga (Bhongade *et al*., 2015; Yao & Mills, 2016). Further study is needed to examine the impact of stress-reduction strategies and treatments like cognitive behavioral therapy and mindfulness on mental health outcomes (Yao & Mills, 2016). The effects of stress on sexual performance and fertility must also be considered and managed. Adequate sleep appears to be an essential element that may increase the quality of semen (Alvarenga *et al*., 2015). However, there are still many lifestyle characteristics for which thresholds have not been established, necessitating more research.

## RECOMMENDATIONS AND CONCLUSIONS

Male infertility is a major health challenge all over the globe today with many cases linked to lifestyle-related factors. The quality of sperm is largely impacted by obesity, addiction to recreational drugs, extensive exposure to radiation-emitting gadgets, and stress. Sperm count, motility, morphology, and DNA damage are all compromised by lifestyle factors, which may also affect the endocrine regulation of reproductive functions. A couple’s desire for a child will be more successful if other contributing elements such as genetic factors, clinical considerations, and work and environmental factors are also taken into consideration. The holistic view of these different factors could provide a better understanding of how best to prevent or at least mitigate the issues that might raise the likelihood of a more favorable conception, pregnancy, and live birth rate for both partners. Men and women alike need to be aware of these possible reasons for infertility to better understand their reproductive potential. When faced with problems affecting fertility, education is essential to recognizing the need for and facilitating therapeutic care. The do’s and don’ts of fertility are equally crucial information. It is not a matter of “increasing” fertility; rather, it is a matter of ensuring that the fertility potential is not prematurely deteriorated so that conception has a fighting chance. Personal self-awareness allows the opportunity to identify several risk factors that represent a threat to fertility. Most of the criteria can be changed with a little deliberate effort to replace bad behaviors with better ones. This may not ensure a good pregnancy, but the health benefits of changing one’s lifestyle are undeniable.

This study, however, advocates employing attention in counseling aged couples who desire to conceive through ART; they should be quickly reassured to minimize their exposure to these factors. Many studies have shown that antioxidants can improve sperm function in individuals with idiopathic male infertility by correcting oxidative stress-induced sperm malfunction. To acquire lifestyle suggestions and the potential use of nutraceutical antioxidants, males should be urged to see an andrologist. A better quality of life for couples’ chances of conceiving a child may be predicted by correcting their lifestyles and reducing the need for costly and intrusive reproductive therapies.
